# Synbiotics as Treatment for Irritable Bowel Syndrome: A Review

**DOI:** 10.3390/microorganisms12071493

**Published:** 2024-07-21

**Authors:** Henning Sommermeyer, Jacek Piątek

**Affiliations:** Department of Health Sciences, Calisia University, Nowy Swiat 4, 62-800 Kalisz, Poland; drpiatek@interia.eu

**Keywords:** gut microbiota, irritable bowel syndrome, probiotics, randomized clinical trials, synbiotics

## Abstract

Irritable bowel syndrome is a persistent disturbance of the function of the gastrointestinal tract with a prevalence of about 11.2% in the population at large. While the etiology of the disorder remains unclear, there is mounting evidence that the disturbance of the gut microbiota is at least one contributing factor. This insight resulted in clinical trials investigating the therapeutic effects of products containing probiotic microorganisms. Most studies with IBS patients have evaluated the therapeutic effects of mono- and multi-strain probiotics, but only a few studies have investigated the efficacy of synbiotics (combinations of probiotic bacteria and one or more prebiotic components). This review summarizes the results from eight randomized, placebo-controlled clinical trials that investigated the efficacy of synbiotic preparations (three mono-strain and five multi-strain products) in adult IBS patients. While data remain sparse, some of the surveyed clinical trials have demonstrated interesting efficacy results in IBS patients. To allow a judgment of the role played by synbiotics in the treatment of IBS patients, more high-quality clinical trials are needed.

## 1. Introduction

Irritable bowel syndrome (IBS) patients suffer from recurrent abdominal pain, bloating, and alterations in the form and frequency of their stool [1]. The disease is benign but impacts patients’ quality of life and work productivity [2,3]. Typical comorbidities of IBS are anxiety and depression [4]. IBS patients cause significant healthcare costs as they frequently utilize healthcare services [5,6]. IBS diagnosis is exclusively based on assessing typical IBS symptoms, and so far, no dependable biological marker is known [7,8,9]. An assessment of the predominant stool pattern with the Bristol Stool Form scale is used to segregate IBS patients into IBS-D (mainly diarrhea), IBS-C (mainly constipation), IBS m (diarrhea and constipation), or IBS-U (insufficient abnormality of stool) subtypes [10]. The prevalence of IBS in the population varies among regions and depends on the diagnostic criteria employed but is, in general, about 11.2% [2,11]. IBS is more frequently diagnosed in females than in males [12].

The cause of IBS is still poorly understood, and environmental, inherited, and psychosocial elements are most likely involved. A variety of mechanisms are discussed as disease-relevant, including the following among them: visceral hypersensitivity, the disturbed function of the gut–brain axis, alterations in the permeability of the gut epithelium, disturbance of gut motility, increased immune system activity, the malfunction of signaling mechanisms of the enteroendocrine system and a disturbed balance of the gut microbiota [13,14]. That a dysbiosis of the bacterial community living in the gut might cause or contribute to the manifestation of IBS has recently become a focus area of IBS research. Comparing the gut microbiota of IBS patients with those of healthy controls revealed significant differences in their composition [15,16,17]. In addition, it was found that certain gut microbiota profiles correlate with specific IBS symptom patterns and the severity of the disease [18,19]. Not surprisingly, an increasing number of publications have reported results of clinical trials investigating the potential therapeutic benefits of probiotics, prebiotics, and synbiotics [20].

Compared to earlier reviews on the subject, the present review is purely focused on clinical trials investigating the effects of synbiotics in adult IBS patients. It is the first review including data from a recently published clinical trial characterizing a balanced nine-strain synbiotic, which so far is the largest IBS trial characterizing preparations from this product category. In addition, this trial is the first that reports the beneficial effects of a tested synbiotic across all major IBS symptoms.

## 2. Methods

This review does not aim to perform a systematic review but to provide an overview of the clinical designs and results of randomized, double-blind, placebo-controlled clinical trials, evaluating the effects of treatment with synbiotic preparations in adult patients with IBS. Based on the findings of this review, a set of recommendations for future clinical trials investigating synbiotics in IBS patients have been made. The PubMed Central database was searched up to 31 May 2024 to identify randomized, double-blind, placebo-controlled clinical trials reporting treatment effects of synbiotics in adult IBS patients. The search strategy comprised six steps. Step 1: Using the advanced search function of the database for the following term combinations: “randomized” and “irritable bowel syndrome” and “synbiotic” or “symbiotic” or “probiotic” were searched for in “any field”. This resulted in a list of 1331 publications. Step 2: Elimination of all publications with the term “review” in the title (n = 132). Step 3: From the remaining 1199 publications, those with the term “probiotic” in the title were eliminated, but not when the title contained the additional terms “synbiotic” or “symbiotic” (n = 7) or “prebiotic” and “probiotic” (n = 25). This resulted in 723 remaining publications. Step 4: The titles of these publications were searched for the terms “clinical trial” or “trial” or “clinical study” or “study” or “randomised”, which resulted in the identification of 24 publications. Step 5: The abstracts of these publications were analyzed, which resulted in the elimination of 16 publications. Reasons for excluding publications were as follows: (i) open-label studies (n = 4), (ii) studies not placebo-controlled (n = 2), (iii) studies not double-blinded (n = 1), (iv) the tested product was not synbiotic with living bacteria (n = 2), (v) no IBS patient was investigated (n = 1), (vi) children with IBS were investigated (n = 1), or (vii) other reasons (study protocol, commentary, animal study, study evaluating fecal microbiota, or a publication in Russian language with an uncomprehensive abstract in English) (n = 5). Step 6: The remaining eight publications underwent a full-text analysis. A flow diagram of the search process is provided as Supplementary Material.

## 3. Supplementation of IBS Patients with Probiotics

In 2014, an expert consensus document defined probiotics as living microorganisms (yeasts, or more commonly, bacteria), which, when administered in adequate amounts, conferred a health benefit on the host [21]. The beneficial effects of probiotic bacteria seem to be more related to specific bacterial strains than bacterial species. The number of bacteria in a probiotic preparation is normally provided as the number of colony-forming units (cfu).

The administration of products containing probiotic bacteria has been shown to normalize disturbed gut microbiota and is therefore considered a therapeutic option for the treatment of IBS patients. Several publications report the results from randomized clinical trials that investigate the therapeutic effects of single- or multi-strain probiotics, indicating that the investigated probiotic products have beneficial effects on at least some of the IBS symptoms [22]. However, recently published meta-analyses and systematic reviews of these trials came to the conclusion that a generalization of the obtained results is a challenging task [20,23,24,25,26]. There are a number of reasons why the analysis of these trial results is so difficult: (i) there are a variety of tested probiotic products with few evaluated in at least two independent clinical trials, (ii) the heterogeneity of clinical trial designs has little standardization of clinical endpoints, (iii) the often small number of trial participants, and (iv) the sometimes short treatment duration. Nevertheless, the currently available clinical trial data for probiotics indicate that certain members of this product category reduce the severity of some IBS symptoms in certain types of IBS patients. These beneficial effects seem to be more dependent on particular bacterial strains than the bacterial species contained in the products. Whether multi-strain probiotics exhibit superior efficacy in IBS patients compared to mono-strain probiotics remains an open question [24,27,28,29].

## 4. Synbiotics as Treatment for IBS Patients

Synbiotics are preparations containing one (single-strain synbiotic) or several (multi-strain synbiotic) probiotic microorganisms combined with one or more prebiotic components [30]. Prebiotics are non-digestible food ingredients that promote the growth of beneficial microorganisms in the intestine [31,32]. Fructooligosaccharides (FOSs), galactooligosaccarides (GOSs), and inulin are important prebiotics used in synbiotic preparations.

Until now, eight publications of placebo-controlled, double-blind, randomized clinical studies have reported the effects of synbiotic preparations on IBS symptoms in adult patients. Of these eight trials, three investigated single-strain synbiotics [33,34,35], while the remaining five trials studied the effects of multi-strain synbiotics containing 5 to 9 different probiotic strains [36,37,38,39,40]. Treatment durations varied between 2 and 12 weeks. Only three trials comprised run-in phases to establish baseline values for the assessed measures [37,39,40]. The number of trial participants (randomized patients) ranged from 68 to 202.

One of the single-strain trials characterized a synbiotic preparation of *Bifidobacterium animalis subsp*. *lactis* Bb-12 and acacia fiber in IBS patients (n = 130) [33]. After eight weeks of treatment with the symbiotic, a significant beneficial effect on overall IBS symptoms and bowel habit satisfaction was found when compared to the placebo. However, no significant differences between the placebo and the synbiotic group were found when the measurements of abdominal pain/discomfort, abdominal distension/bloating, and quality of life were analyzed.

In the second published single-strain synbiotic trial, adult IBS patients (n = 85) were treated for twelve weeks with a preparation containing *Bacillus coagulans* (though no strain information was provided by authors) and fructooligosaccharides (FOS) [34]. More patients from the synbiotic group than those of the placebo group experienced a reduction in the occurrence rates of abdominal pain and diarrhea. However, the treatment did not change the frequency of constipation. In contrast to many other studies with probiotics or synbiotics, a high number of patients taking the synbiotic experienced adverse events, which resulted in a 41% dropout rate in this treatment group (compared to 25% in the placebo group).

In the third single-strain synbiotic trial, 67 elderly IBS patients (age ≥ 60 years) were treated for four weeks with a mixture of *Lactobacillus paracasei* DKGF1 (named according to current taxonomy: *Lacticaseibacillus paracasei*) and the prebiotic *Opuntia humifusa* [35]. Treatment with the synbiotic resulted in a higher overall responder rate, reduced abdominal pain, and improved psychological well-being compared to treatment with a placebo. No significant differences in gas or bloating symptoms were observed.

A study with 126 IBS patients characterizing the effect of a multi-strain synbiotic containing seven probiotic strains was published by Shavakhi et al. in 2013 [36]. The probiotic bacteria contained in the synbiotic preparation were as follows: *Lactobacillus casei* (named according to current taxonomy: *Lacticaseibacillus casei*), *Lactobacillus rhamnosus* (named according to current taxonomy: *Lacticaseibacillus rhamnosus*), *Lactobacillus acidophilus*, *Lactobacillus bulgaricus* (named according to current taxonomy: *Lactobacillus delbrueckii subsp. bulgaricus*), *Bifidobacterium breve*, *Bifidobacterium longum*, and *Streptococcus thermophilus* (though no strain information was provided by authors). The mixture of probiotic bacteria was combined with FOS as a prebiotic component. There were no significant differences between the synbiotic and the placebo group found when analyzing the measurements of abdominal pain and distension. However, the failure to show the superiority of the synbiotic might have been caused by the short treatment duration (two weeks) evaluated in the trial.

Results of a four-week trial evaluating the effects of a nine-strain synbiotic containing six lactobacilli, two bifidobacteria, and *Streptococcus thermophilus* were published by Cappello et al. in 2013 [37]. Probiotic bacteria contained in the synbiotic preparation were as follows:

*Lactobacillus plantarum* (named according to current taxonomy: *Lactiplantibacillus plantarum subsp. plantarum*), *Lactobacillus casei subp. rhamnosus* (named according to current taxonomy: *Lacticaseibacillus rhamnosus*), *Lactobacillus gasseri*, *Lactobacillus acidophilus*, *Lactobacillus salivarius* (named according to current taxonomy: *Ligilactobacillus salivarius*), *Lactobacillus sporogenes* (named according to current taxonomy: *Heyndrickxia coagulans*), *Bifidobacterium infantis*, and *Bifidobacterium longum* (though no strain information was provided by the authors) The prebiotic component of the preparation was inulin. A total of 64 IBS patients were included in the trial, and a significant decrease in the severity of abdominal flatulence was found when the symbiotic group was compared with the placebo group. However, treatment with the synbiotic failed to improve the global satisfactory relief of abdominal flatulence and bloating. The study also investigated the severity of pain and urgency on a weekly basis but found no significant differences between the placebo and the synbiotic group.

In 2019, a study was published characterizing the dose-dependent effects of a synbiotic containing six lactobacilli and two bifidobacteria [38]. The bacteria mixture comprised the following: *Lactobacillus rhamnosus* (named according to current taxonomy: *Lacticaseibacillus rhamnosus*), *Lactobacillus acidophilus*, *Lactobacillus casei* (named according to current taxonomy: *Lacticaseibacillus casei*), *Lactobacillus bulgaricus* (named according to current taxonomy: *Lactobacillus delbrueckii subsp. bulgaricus*), *Lactobacillus plantarum* (named according to current taxonomy: *Lactiplantibacillus plantarum subsp. plantarum*), *Lactobacillus salivarius* (named according to current taxonomy: *Ligilactobacillus salivarius*), *Bifidobacterium bifidum*, and *Bifidobacterium longum* (though no strain information was provided by the authors). The probiotic bacteria mixture was combined with a mixture of four prebiotic compounds (FOS, inulin, *Geum urbanum*, and *Ulmus davidiana*). The results from 28 IBS patients were analyzed, revealing significant beneficial effects after 8 weeks of treatment with a high dose of the synbiotic for the measures of fatigue, abdominal discomfort, bloating, and the occurrence rate of normal stools.

Skrzydlo-Radomanska et al. published results from a study involving 68 IBS-D patients treated for 8 weeks with a synbiotic containing five probiotic strains (two lactobacilli and three bifidobacteria) and the prebiotic FOS [39]. Probiotic strains in the synbiotic were as follows: (i) *Lactobacillus rhamnosus* FloraActiveTM 19070-2 (named according to current taxonomy: *Lacticaseibacillus rhamnosus*), *Lactobacillus acidophilus* DSMZ 32418, *Bifidobacterium lactis* DSMZ 32269, *Bifidobacterium longum* DSMZ 32946, and *Bifidobacterium bifidum* DSMZ 32403. Measurements for the severity of IBS symptoms using the IBS-Severity of Symptoms Scale (IBS-SSS) revealed a significant decrease in IBS symptom severity in the synbiotic group when compared with the placebo group. The effect was mainly driven by an improvement in the severity of flatulence (IBS-SSS3 subscale) with no effect on the perception of pain. After 8 weeks of treatment, patients in the synbiotic treatment group showed a significant improvement on the IBS-Global Improvement Scale (IBS-GIS) when compared to the placebo group. Comparing the results of adequate relief measurements using the IBS-adequate relief Scale (IBS-AR), no differences of significant importance were revealed between the two treatment groups.

Most recently, results from a study that involved 202 IBS patients across various stool form types and disease severity levels were published [40]. Patients were treated for 12 weeks with a balanced nine-strain synbiotic (four *Lactobacilli*, *Lactococcus lactis*, three *Bifidobacteria*, *Streptococcus thermophilus*) and the prebiotic FOS. The probiotic strains contained in the synbiotic preparation were as follows: *Lactobacillus helveticus* SP 27, *Lacticaseibacillus rhamnosus* Lr-32, *Lacticaseibacillus casei* Lc-11, *Lactiplantibacillus plantarum* Lp-115, *Lactococcus lactis* Ll-23, *Bifidobacterium longum* Bl-05, *Bifidobacterium breve* Bb-03, *Bifidobacterium bifidum* Bb-02, and *Streptococcus thermophilus* St-21. Compared to the placebo group, treatment with the synbiotic resulted in significant beneficial effects across all major symptoms of IBS. After 12 weeks of treatment, 98% of the patients in the multi-strain synbiotic group and 14% of those treated with a placebo experienced a decrease in IBS symptom severity by at least 50 points on the IBS-SSS. Of the patients treated with the symbiotic, 89% showed an improvement in the IBS-GIS by at least two points (0% of the placebo patients). For 70% of the patients taking the symbiotic, adequate relief (using the IBS-AR scale) was determined (for 0% of the placebo patients). In addition, patients treated with the synbiotic self-reported reductions in abdominal pain severity, bloating severity, rectal pressure and perceptions of incomplete bowel movements. Of the IBS-D patients (n = 73) treated with the synbiotic, 78% experienced the normalization of their stool form (determined by using the Bristol Stool Form Scale). Of the IBS-D patients receiving a placebo (n = 73), only 3% reported a similar normalization of their stool form. Stool form normalization was also observed in 96% of IBS-C patients treated with the synbiotic (n = 26), while only 10% of IBS-C patients treated with the placebo (n = 20) reported a normalization of their stool form. The only reported adverse events were two cases of transient headaches observed in the synbiotic treatment group. None of the two affected patients decided to prematurely terminate their participation in the clinical trial.

As the study designs and clinical endpoints of clinical trials investigating the effects of synbiotic preparations in IBS patients vary substantially, it is very difficult to summarize their results. An overview of the key clinical trial design features and outcomes is provided in Table 1.

Among the eight clinical studies that evaluated synbiotics in IBS patients, only one failed to demonstrate beneficial effects in patients. However, this may have been caused by the very short treatment duration of only two weeks, which was investigated in this particular clinical trial [36]. In most of the studies with positive outcomes, the improvements covered at least some of the main IBS symptoms. So far, the only synbiotic exhibiting a broad range of beneficial effects for all types of IBS symptoms is the one investigated in the recently published trial evaluating the effects of a balanced nine-strain synbiotic [40]. Analyzing data from 201 IBS patients, this study is, to date, the largest published clinical trial investigating the effects of a synbiotic preparation on IBS symptoms. The study was performed in two clinical centers, placebo-controlled, double-blind, and allocated patients to the two treatments randomly. This study, therefore, fulfills the major requirements of a good quality clinical trial design. The 12-week treatment duration after a four-week treatment-free run-in phase and the use of established scales (IBS-SSS, IBS-GIS, and IBS-AR) as study endpoints add to the quality of this study.

The present review does not aim for an in-depth analysis of the respective certainty of evidence (GRADE) for each individual trial. However, as there are clear differences in the quality of the studies, some “risk of bias assessment” for the overall quality of the trials is provided in Table 2. Only four of the analyzed publications specified the probiotic strains contained in the synbiotics, which were investigated in the particular clinical trial [33,35,39,40]. In the other publications, the authors only provided information about the bacterial species in the synbiotic tested [34,36,37,38]. As the effects of probiotic bacteria are strain- rather than species-specific, this lack of information is problematic when interpreting the trial results.

While all trials included in the analysis are randomized, double-blinded, and placebo-controlled, not all of them have been registered in clinical trial registries [33,37,38]. The registration of the clinical trial published by Oh et al. in 2022 [35] was performed only after enrollment completion. Already in 2004, the International Committee of Medical Journal Editors (ICMJEs) requested that all clinical trials starting enrollment after 1 July 2005 should be registered in a public trial registry before the time of first patient enrollment as a condition of consideration for publication [41]. As all four trials were performed and published significantly later than 2005 (the oldest publication [33] was from 2012), pre-trial registration should have been conducted for all of them. This represents a major quality concern as proper pre-trial registration is considered mandatory to avoid the selective reporting of trial results. Nevertheless, the authors decided not to eliminate these four trials from the review. While the trials are of questionable quality, the published results might add to the still very limited data available characterizing the effects of synbiotics in IBS patients. However, the results of these four trials have to be interpreted with great care, as the reported results might represent only a selected set of data. Only one trial is a multi-center trial [40], with the publication of another trial [39] not being clear in this regard. More recently published trials make use of established scales used in clinical IBS research (e.g., IBS-SSS, IBS-GIS, or IBS-AR) as clinical endpoints, which make the interpretation of results easy.

Theoretically, synbiotics should have an advantage over probiotics as they carry a source of energy (the prebiotic component) for probiotic bacteria, supporting their proliferation and the colonization of the intestine of the host. To prove if this potential advantage manifests in reality, one would need to compare a synbiotic with the corresponding probiotic component (without the prebiotic component) in a head-to-head study. So far, no such study has been published. Alternatively, one could compare independent studies, one investigating a given synbiotic and a second investigating its probiotic component. However, for the eight synbiotics covered by this review, we were not able to find studies of matching study designs characterizing the corresponding probiotic components. Finally, currently, available data are too sparse to evaluate if the kind of prebiotic component contained in synbiotics plays a major role in their effects on IBS patients.

## 5. Summary and Conclusions

Data from randomized clinical trials evaluating the efficacy of synbiotics in IBS patients are still sparse. The number of publications reporting the efficacy data of synbiotics obtained in clinical trials with IBS patients is growing, which is a fact indicating that this product category is a promising area for future research. Balanced multi-strain synbiotics might be especially interesting candidates for future investigations. However, this research will need to employ high-quality clinical study designs, large enough numbers of trial participants, long enough treatment durations, and effect confirmation through independent confirmatory trials. The publications of study results should contain information about the probiotic strains contained in the tested synbiotic preparations using the current taxonomy for the bacteria. It should also be generally accepted that the registration of any future clinical trial in a clinical trial registry (e.g., ClinicalTrials.gov) before the start of enrollment is mandatory.

## Figures and Tables

**Table 1:** Summary of trial design features and key study results. Data refer to patients analyzed if not otherwise stated.

Publication [Reference]	Study Type	Participants f/m IBS Subtypes	Number of Participants ^1^ Randomized (Analyzed) Adverse Events	Preparation/Dosing [Trademark]	Probiotic Component Total Daily cfu Dose (Enteric Coating? ^2^)	Prebiotic(s) Component Total Daily Dose	Run-in Phase/ Treatment Phase	Primary Endpoints	Secondary Endpoints
Min et al., 2012 [33]	single-center randomized double-blind placebo-controlled	Rome III criteria for IBS f 82/m 35 IBS-D: 35 IBS-C: 41 IBS-M: 10 IBS-U: 31	T: 130 (117) P: 65 (59) S: 65 (58) AE: none	Standard yogurt enriched with acacia dietary fiber and *Bifidobacterium lactis* Bb-12 (≥10^11^ cfu/bottle)/ two bottles of 150 mL per day[none]	≥200 × 10^9^ cfu (no)	Acacia fiber (no information about amount)	0 weeks/ 8 weeks	Overall IBS symptoms in S group patients with IBS-C significantly reduced compared to P. Bowel habit satisfaction in S group patients with IBS-D significantly improved compared to P.	Better improvements in abdominal pain/discomfort, abdominal distention/bloating, discomfort related to daily life, and straining in S group compared to P, but these were not statistically significant. No difference between the two groups for straining or feelings of incomplete evacuation
Rogha et al., 2014 [34]	single-center randomized double-blind placebo-controlled	Rome III criteria for IBS f 44/m 12 IBS-D: 18 IBS-C: 7 IBS-M: 23 IBS-U: 3	T: 85 (56) P: 44 (23) S: 41 (23) AE: 17 in S group and 11 in P group	*Bacillus coagulans* (15 × 10^7^ spores/tablet), FOS/ three tablets per day [Lactol^®^, Natures Only, Inc. Villa Park, CA, USA]]	0.45 × 10^9^ spores (no)	FOS 300 mg	0 weeks/ 12 weeks	A significantly higher reduction in abdominal pain frequency in the S group compared to P. Diarrhea frequency reduced in S group but not in P group. Decrease in constipation frequency same in both groups.	none
Oh et al., 2022 [35]	single-center randomized double-blind placebo-controlled	Rome IV criteria for IBS f 48/m 21 IBS-D: 9 IBS-C: 7 IBS-M: 5 IBS-U: 46	T: 68 (67) P: 34 (34) S: 34 (33) AE: none	*Lactobacillus paracasei* DKGF1 (10^11^ cfu), extracts of Opuntia humifusa/ one sachet per day [none]	100 × 10^9^ cfu (no)	Opuntia humifusa 200 mg	0 weeks/ 2 weeks	SGA: Responder rate (>2 points), P 23.5%, S (51.5%) (s) VSA: Abdominal pain P 81.8%, S 58.8% (s),psychological well-being P 26.4%, S 60.6% (s). No significant differences in gas or bloating symptoms.	BSFS: Responder rate (improvement in stool form and frequency) higher in S than in P (s)
Shavakhi et al., 2013 [36]	single-center randomized double-blind placebo-controlled	Rome II criteria for IBS f 85/m 44 IBS-D: 42 IBS-C: 59 IBS-M: 28	T: 132 (129) P: 66 (63) S: 66 (66) AE: 3 in S group	*Lactobacillus casei*, *Lactobacillus rhamnosus*, *Lactobacillus acidophilus*, *Lactobacillus bulgaricus*, *Bifidobacterium breve*, *Bifidobacterium longum*, *Streptococcus thermophilus*, total 0.1 × 10^9^ cfu/capsule, FOS/ two capsules per day [Balance^®^, Protexin Co., Sommerset, UK]	0.2 × 10^9^ cfu (no enteric coating)	FOS (no information about amount)	0 weeks/ 2 weeks	Abdominal pain: Decrease in P and S with no significant difference. Improvement in bowel: P 36.5%, S 33.3% (NS)	QoL: No significant difference between P and S
Capello et al., 2013 [37]	single-center randomized double-blind placebo-controlled	Rome III criteria for IBS f 41/m 23 IBS-D: 23 IBS-C: 25 IBS-M: 14 IBS-U: 2	T: 68 (64) P: 34 (32) S: 34 (32) AE: none	*Lactobacillus plantarum* (5 × 10^9^), *Lactobacillus casei*, *subp. rhamnosus* (2 × 10^9^), *Lactobacillus gasseri* (2 × 10^9^), *Bifidobacterium infantis* (1 × 10^9^), *Bifidobacterium longum* (1 × 10^9^), *Lactobacillus acidophilus* (1 × 10^9^), *Lactobacillus salivarius* (1 × 10^9^), *Lactobacillus sporogenes* (1 × 10^9^), *Streptococcus thermophilus* (5 × 10^9^), inulin/ two sachets per day [Probinulin, CaDi Group, Rome, Italy]	36 × 10^9^ cfu (no)	Inulin 4.400 mg	2 weeks/ 4 weeks	Responder rates (pts reported during 50% of treatment weeks included a global satisfactory relief of) abdominal flatulence and bloating: P 65.6% and S 46.9% (ns). Responder rate flatulence: P 62.5% and S (ns).	Score of flatulence significantly lower in S group compared to P. No differences between the two groups for symptoms of bloating, pain, urgency, bowel function or feelings of incomplete evacuation.
Lee et al., 2019 [38]	single-center randomized double-blind placebo-controlled	Rome III criteria for IBS f 15/m 13 IBS-D: 20 IBS-C: 6 IBS-M: 2	T: 30 (28) P: 10 (10) S low:10 (10) S high 10 (8) AE: none	*Lactobacillus rhamnosus*, *Lactobacillus acidophilus*, *Lactobacillus casei*, *Lactobacillus bulgaricus*, *Lactobacillus plantarum*, *Lactobacillus salivarius*, *Bifidobacterium bifidum*, *Bifidobacterium longum*, total of 10 × 10^9^ cfu/capsule, FOS, Ulmus davidiana, Geum urbanum, inulin/ once daily [Ultra Probiotics 500, B&A Health Products Inc., Los Angele, CA, USA]	10 × 10^9^ cfu (no)	FOS 175 mg, *Ulmus davidiana* 150 mg, *Geum urbanum* 10 mg, Inulin 100 mg	0 weeks/ 8 weeks	Abdominal discomfort, abdominal bloating, frequency of formed stool, and fatigue VAS significantly improved in the S high-dose group compared to P.	None
Skrzydlo-Rado- manska et al., 2020 [39]	multi-center ^3^ randomized double-blind placebo-controlled	Rome III criteria for IBS and BSFS scores 5-7 f 49/m 19 IBS-D: 68	T: 80 (68) P: 40 (33) S: 40 (35) AE: 4 in S group and 3 in P group	*Lactobacillus rhamnosus* FloraActiveTM 19070-2 (9.8 × 10^8^), *Lactobacillus acidophilus* DSMZ 32,418 (4.9 × 10^8^), *Bifidobacterium lactis* DSMZ 32,269 (29.4 × 10^8^), *Bifidobacterium longum* DSMZ 32,946 (2.94 × 10^8^), *Bifidobacterium bifidum* DSMZ 32,403 (2.94 × 10^8^), total amount of cfu 50 × 10^8^/sachet), scFOS/ two sachets per day [none]	10 × 10^9^ cfu (no)	scFOS 1.914 mg	2 weeks/ 8 weeks	IBS-SSS responder rate: after 4 weeks P 3.0%; S 22.9% (s), after 8 weeks P 21%, S 40% (ns). Number of patients with IBS-GIS score ˃4: after 8 weeks in S larger than P (ns) IBS-AR: no difference between P and S IBS-QoL: no difference between P and S	Significant amelioration of feelings of incomplete stool evacuation, flatulence, pain, stool pressure and diarrheal stools after 8 weeks of treatment.
Sommer- meyer et al., 2024 [40]	multi-center randomized double-blind placebo-controlled	Rome III criteria for IBS (questionnaire for HCP of WGO) f 119/m 82 IBS-D: 146 IBS-C: 46 IBS-M: 3 IBS-U: 6	T: 202 (201) P: 101 (100) S:101 (101) AE: 2 in S group	*Lactobacillus helveticus* SP 27 (9 × 10^8^) *Lacticaseibacillus rhamnosus* Lr-32 (4.5 × 10^8^), *Lacticaseibacillus casei* Lc-11 (2.25 × 10^8^), *Lactiplantibacillus plantarum* Lp-115 (2.25 × 10^8^), *Lactococcus lactis* Ll-23 (9 × 10^8^), *Bifidobacterium longum* Bl-05 (6.75 × 10^8^), *Bifidobacterium breve* Bb-03 (4.5 × 10^8^), *Bifidobacterium bifidum* Bb-02 (2.25 × 10^8^) *Streptococcus thermophilus* St-21 (4.5 × 10^8^), FOS/ one capsule per day [Vivatlac^®^ Synbiotikum, Vivatrex GmbH, Rees, Germany]	4.5 × 10^9^ cfu (yes)	FOS 63 mg	4 weeks/ 12 weeks	IBS-SSS responder rate: after 12 weeks P 14%, S 98% (s) IBS-GIS improvement by 3 (2) points after 12 weeks P 0% (0%), S 52.5% (89.1%) (s) Adequate relief (IBS-AR) after 12 weeks achieved P 0%, S 70%. (s)	Significant improvements in S compared to P achieved for the severity of abdominal pain, severity of flatulence, stool pressure, and feelings of incomplete evacuation. Stool normalization in 78.1% of IBS-D and 96.2% of IBS-C patients after 12 weeks of treatment

Names of probiotic bacteria in the table are taken from the original publication. In some cases, these names do not reflect the current taxonomy. In the main text of the present publication the actual names of bacteria are provided in brackets after the name used in the original publication. Four of the reviewed publications [34,36,37,38] do not provide strain-specific information for probiotic bacteria in the tested synbiotics. Abbreviations used in the table: AE: adverse events; BSFS: Bristol Stool Form Scale; cfu: colony-forming units; FOS: fructooligosaccharides; IBS: irritable bowel syndrome IBS-AR: IBS—adequate relief scale; IBS-C: IBS with predominant constipation; IBS-D: IBS with predominant diarrhea; IBS-GIS: IBS—Global Improvement Scale; IBS-M: IBS with diarrhea and constipation; IBS-QoL: IBS—Quality of Life scale; IBS-SSS: IBS—Severity of Symptoms Scale; IBS-U: unspecified IBS; (ns): no significant difference between P- and S-group P: placebo group; (s): significant difference between P- and S-group S: synbiotic group; scFOS: short-chain fructooligosaccharides S high: high-dose symbiotic S; low: low-dose symbiotic SGA: Subjective Global Assessment Scale; T: total; VAS: Visual Analog Scale ^1^ Percentages are the % of all patients in the respective group; ^2^ Enteric coating refers to information about the protection of the probiotic bacteria in the synbiotic against inactivation by stomach acid ^3^ Unclear, publication states “performed at outpatient clinics”.

**Table 2:** Risk of bias assessment for the overall quality of analyzed clinical trials.

Publication [Reference]	Trial Registration ^1^	Number of Study Centers	Sequence Generation	Allocation Concealment	Blinding	Placebo Description (Content)	Loss to Follow-Up ^2^	Registered Primary Endpoints	Registered Secondary Endpoints
Min et al., 2012 [33]	none	1	random number table	no details provided	double-blind	yes (non-enriched yogurt)	T: 13 (10.0%) P: 6 (9.2%) S: 7 (10.8%)	none registered before the trial start	none registered before the trial start
Rogha et al., 2014 [34]	ClinicalTrials.gov NCT01837485	1	no details provided	no details provided	double-blind	yes (lactose starch, tartrazine)	T: 29 (34.1%) P: 11 (24.4%) S: 18 (43.9%)	abdominal pain constipation diarrhea	none
Oh et al., 2022 [35]	Cris.Nih.Go.Kr KCT0005449 (registration after start of enrollment)	1	software generated randomization list	no details provided	double-blind	yes (maltodextrin)	T: 1 (1.4%) P: 0 (0.0%) S: 1 (2.9%)	SGA ^4^ VAS ^5^	Bristol Stool Form Scale
Shavakhi et al., 2013 [36]	ClinicalTrials.gov NCT01837472	1	random number table	no details provided	double-blind	yes (not specified)	T: 3 (2.3%) P: 3 (4.5%) S: 0 (0.0%)	abdominal pain	QoL
Capello et al., 2013 [37]	none	1	block randomization	no details provided	double-blind	yes (not specified)	T: 4 (5.9%) P: 2 (5.9%) S: 2 (5.9%)	none registered before the trial start	none registered before the trial start
Lee et al., 2019 [38]	none	1	no details provided	no details provided	double-blind	yes (not specified)	T: 2 (6.7%) P: 0 (0.0%) S low: 0 (0%) S high 2 (20%)	none registered before the trial start	none registered before the trial start
Skrzydlo-Radomanska et al., 2020 [39]	ClinicalTrials.gov NCT04206410	? ^3^	software generated randomization list	allocated by a physician based on randomization list	double-blind	yes (maltodextrin)	T: 12 (15.0%) P: 7 (17.5%) S: 5 (12.5%)	IBS-SSS ^6^ IBS-GIS ^7^ IBS-AR ^8^ IBS-QoL ^9^	BSFS ^10^, pain, flatulence, feelings of incomplete evacuation, and number of bowel movements
Sommermeyer et al., 2024 [40]	ClinicalTrials.gov NCT05731232	2	software generated randomization	allocated by physician based on randomization list and entrance sequence	double-blind	Yes (maize starch)	T: 1 (0.5%) P: 1 (1.0%) S: 0 (0.0%)	IBS-SSS IBS-GIS IBS-AR	BSFS, pain, bloating, stool pressure, feelings of incomplete evacuation, and number of bowel movements

^1^ URL of registry followed by registration number of the trial; ^2^ T: total, P: placebo, S: synbiotic, numbers represent the number of lost patients, percentages are the % of all patients in the respective group; ^3^ unclear, publication states “performed at outpatient clinics”; ^4^ Subjective Global Assessment Scale; ^5^ Visual Analog Scale; ^6^ IBS-Severity of Symptoms Scale, ^7^ IBS-Global Improvement Scale; ^8^ IBS-adequate relief scale, ^9^ IBS-Quality of Life scale; ^10^ Bristol Stool Form Scale.

## Supplementary Materials

The following supporting information can be downloaded at: https://www.mdpi.com/article/10.3390/microorganisms12071493/s1, Figure S1: Flow diagram of the search strategy employed for the analysis.

## References

[1] Ford A.C., Sperber A.D., Corsetti M., Camilleri M. (2020). Irritable bowel syndrome. Lancet.

[2] Black C.J., Ford A.C. (2020). Global burden of irritable bowel syndrome: Trends, predictions and risk factors. Nat. Rev. Gastroenterol. Hepatol..

[3] Camilleri M. (2021). Diagnosis and Treatment of Irritable Bowel Syndrome: A Review. JAMA.

[4] Drossman D.A., Chang L., Schneck S., Blackman C., Norton W.F., Norton N.J. (2009). A focus group assessment of patient perspectives on irritable bowel syndrome and illness severity. Dig. Dis. Sci..

[5] Tornkvist N.T., Aziz I., Whitehead W.E., Sperber A.D., Palsson O.S., Hreinsson J.P., Simrén M., Törnblom H. (2021). Health care utilization of individuals with Rome IV irritable bowel syndrome in the general population. United European Gastroenterol. J..

[6] Shah E.D., Salwen-Deremer J.K., Gibson P.R., Muir J.G., Eswaran S., Chey W.D. (2022). Comparing Costs and Outcomes of Treatments for Irritable Bowel Syndrome with Diarrhea: Cost-Benefit Analysis. Clin. Gastroenterol. Hepatol..

[7] Sood R., Law G.R., Ford A.C. (2014). Diagnosis of IBS: Symptoms, symptom-based criteria, biomarkers or ‘psychomarkers’?. Nat. Rev. Gastroenterol. Hepatol..

[8] Francis C.Y., Morris J., Whorwell P.J. (1997). The irritable bowel severity scoring system: A simple method of monitoring irritable bowel syndrome and its progress. Aliment. Pharmacol. Ther..

[9] Drossman D.A., Chang L., Bellamy N., Gallo-Torres H.E., Lembo A., Mearin F., Norton N.J., Whorwell P. (2011). Severity in irritable bowel syndrome: A Rome Foundation Working Team report. Am. J. Gastroenterol..

[10] Blake M.R., Raker J.M., Whelan K. (2016). Validity and reliability of the Bristol Stool Form Scale in healthy adults and patients with diarrhoea-predominant irritable bowel syndrome. Aliment. Pharmacol. Ther..

[11] Lovell R.M., Ford A.C. (2012). Global prevalence of and risk factors for irritable bowel syndrome: A meta-analysis. Clin. Gastroenterol. Hepatol..

[12] Oka P., Parr H., Barberio B., Black C.J., Savarino E.V., Ford A.C. (2020). Global prevalence of irritable bowel syndrome according to Rome III or IV criteria: A systematic review and meta-analysis. Lancet Gastroenterol. Hepatol..

[13] Lacy B.E., Mearin F., Chang L., Chey W.D., Lembo A.J., Simren J., Spiller R. (2016). Bowel disorders. Gastroenterology.

[14] Barbara G., Feinle-Bisset C., Ghoshal U.C., Quigley E.M., Santos J., Vanner S., Vergnolle N., Zoetendal E.G. (2016). The Intestinal Microenvironment and Functional Gastrointestinal Disorders. Gastroenterology.

[15] Tana C., Umesaki Y., Imaoka A., Handa T., Kanazawa M., Fukudo S. (2010). Altered profiles of intestinal microbiota and organic acids may be the origin of symptoms in irritable bowel syndrome. Neurogastroenterol. Motil..

[16] Kerckhoffs A.P., Samsom M., van der Rest M.E., de Vogel J., Knol J., Ben-Amor K., Akkermans L.M. (2009). Lower Bifidobacteria counts in both duodenal mucosa-associated and fecal microbiota in irritable bowel syndrome patients. World. J. Gastroenterol..

[17] Kassinen A., Krogius-Kurikka L., Mäkivuokko H., Rinttilä T., Paulin L., Corander J., Malinen E., Apajalahti J., Palva A. (2007). The fecal microbiota of irritable bowel syndrome patients differs significantly from that of healthy subjects. Gastroenterology.

[18] Tap J., Derrien M., Törnblom H., Brazeilles R., Cools-Portier S., Doré J., Störsrud S., Le Nevé B., Öhman L., Simrén M. (2017). Identification of an Intestinal Microbiota Signature Associated With Severity of Irritable Bowel Syndrome. Gastroenterology.

[19] Malinen E., Krogius-Kurikka L., Lyra A., Nikkilä J., Jääskeläinen A., Rinttilä T., Vilpponen-Salmela T., von Wright A.J., Palva A. (2010). Association of symptoms with gastrointestinal microbiota in irritable bowel syndrome. World. J. Gastroenterol..

[20] Ceccherini C., Daniotti S., Bearzi C., Re I. (2022). Evaluating the Efficacy of Probiotics in IBS Treatment Using a Systematic Review of Clinical Trials and Multi-Criteria Decision Analysis. Nutrients.

[21] Hill C., Guarner F., Reid G., Gibson G.R., Merenstein D.J., Pot B., Morelli L., Canani R.B., Flint H.J., Salminen S. (2014). Expert consensus document. The International Scientific Association for Probiotics and Prebiotics consensus statement on the scope and appropriate use of the term probiotic. Nat. Rev. Gastroenterol. Hepatol..

[22] van der Geest A.M., Schukking I., Brummer R.J.M., van de Burgwal L.H.M., Larsen O.F.A. (2022). Comparing probiotic and drug interventions in irritable bowel syndrome: A meta-analysis of randomised controlled trials. Benef. Microbes..

[23] Niu H.L., Xiao J.Y. (2020). The efficacy and safety of probiotics in patients with irritable bowel syndrome: Evidence based on 35 randomized controlled trials. Int. J. Surg..

[24] Zhang T., Zhang C., Zhang J., Sun F., Duan L. (2022). Efficacy of Probiotics for Irritable Bowel Syndrome: A Systematic Review and Network Meta-Analysis. Front. Cell. Infect. Microbiol..

[25] Chlebicz-Wojcik A., Slizewska K. (2021). Probiotics, Prebiotics, and Synbiotics in the Irritable Bowel Syndrome Treatment: A Review. Biomolecules.

[26] Zhang W.X., Shi L.B., Zhou M.S., Wu J., Shi H.Y. (2023). Efficacy of probiotics, prebiotics and synbiotics in irritable bowel syndrome: A systematic review and meta-analysis of randomized, double-blind, placebo-controlled trials. J. Med. Microbiol..

[27] Dale H.F., Rasmussen S.H., Asiller Ö.Ö., Lied G.A. (2019). Probiotics in Irritable Bowel Syndrome: An Up-to-Date Systematic Re-view. Nutrients.

[28] Liang D., Longgui N., Guoqiang X. (2019). Efficacy of different probiotic protocols in irritable bowel syndrome: A network me-ta-analysis. Medicine.

[29] Xie P., Luo M., Deng X., Fan J., Xiong L. (2023). Outcome-Specific Efficacy of Different Probiotic Strains and Mixtures in Irritable Bowel Syndrome: A Systematic Review and Network Meta-Analysis. Nutrients.

[30] Kolida S., Gibson G.R. (2011). Synbiotics in health and disease. Annu. Rev. Food Sci. Technol..

[31] Gibson G.R., Roberfroid M.B. (1995). Dietary modulation of the human colonic microbiota: Introducing the concept of prebiotics. J. Nutr..

[32] Davani-Davari D., Negahdaripour M., Karimzadeh I., Seifan M., Mohkam M., Masoumi S.J., Berenjian A., Ghasemi Y. (2019). Prebiotics: Definition, Types, Sources, Mechanisms, and Clinical Applications. Foods.

[33] Min Y.W., Park S.U., Jang Y.S., Kim Y.H., Rhee P.L., Ko S.H., Joo N., Kim S.I., Kim C.H., Chang D.K. (2012). Effect of composite yogurt enriched with acacia fiber and Bifidobacterium lactis. World J. Gastroenterol..

[34] Rogha M., Esfahani M.Z., Zargarzadeh A.H. (2014). The efficacy of a synbiotic containing Bacillus Coagulans in treatment of irritable bowel syndrome: A randomized placebo-controlled trial. Gastroenterol. Hepatol. Bed. Bench..

[35] Oh J.H., Jang Y.S., Kang D., Kim H.S., Kim E.J., Park S.Y., Kim C.H., Min Y.W., Chang D.K. (2023). Efficacy of a Synbiotic Containing Lactobacillus paracasei DKGF1 and Opuntia humifusa in Elderly Patients with Irritable Bowel Syndrome: A Randomized, Double-Blind, Placebo-Controlled Trial. Gut Liver.

[36] Shavakhi A., Minakari M., Farzamnia S., Peykar M.S., Taghipour G., Tayebi A., Hashemi H., Shavakhi S. (2014). The effects of multi-strain probiotic compound on symptoms and quality-of-life in patients with irritable bowel syndrome: A randomized placebo-controlled trial. Adv. Biomed. Res..

[37] Cappello C., Tremolaterra F., Pascariello A., Ciacci C., Iovino P. (2013). A randomised clinical trial (RCT) of a symbiotic mixture in patients with irritable bowel syndrome (IBS): Effects on symptoms, colonic transit and quality of life. Int. J. Color. Dis..

[38] Lee S.H., Cho D.Y., Lee S.H., Han K.S., Yang S.W., Kim J.H., Lee S.H., Kim S.M., Kim K.N. (2019). A Randomized Clinical Trial of Synbiotics in Irritable Bowel Syndrome: Dose-Dependent Effects on Gastrointestinal Symptoms and Fatigue. Korean J. Fam. Med..

[39] Skrzydlo-Radomanska B., Prozorow-Krol B., Cichoz-Lach H., Majsiak E., Bierla J.B., Kosikowski W., Szczerbinski M., Gantzel J., Cukrowska B. (2020). The Effectiveness of Synbiotic Preparation Containing Lactobacillus and Bifidobacterium Probiotic Strains and Short Chain Fructooligosaccharides in Patients with Diarrhea Predominant Irritable Bowel Syndrome-A Randomized Double-Blind, Placebo-Controlled Study. Nutrients.

[40] Sommermeyer H., Chmielowiec K., Bernatek M., Olszewski P., Kopczynski J., Piatek J. (2024). Effectiveness of a Balanced Nine-Strain Synbiotic in Primary-Care Irritable Bowel Syndrome Patients-A Multi-Center, Randomized, Double-Blind, Placebo-Controlled Trial. Nutrients.

[41] De Angelis C., Drazen J.M., Frizelle F.A., Haug C., Hoey J., Horton R., Kotzin S., Laine C., Marusic A., Overbeke A.J. (2004). Clinical trial registration: A statement from the International Committee of Medical Journal Editors. Ann. Intern. Med..

