# Supplementary figures and images for: Synbiotics as Treatment for Irritable Bowel Syndrome: A Review
Source: Microorganisms. 2024 Jul 21;12(7):1493. doi: 10.3390/microorganisms12071493 (PMC11278745; doi:10.3390/microorganisms12071493)

**Figure S1.** Flow diagram of the search strategy employed for the analysis.

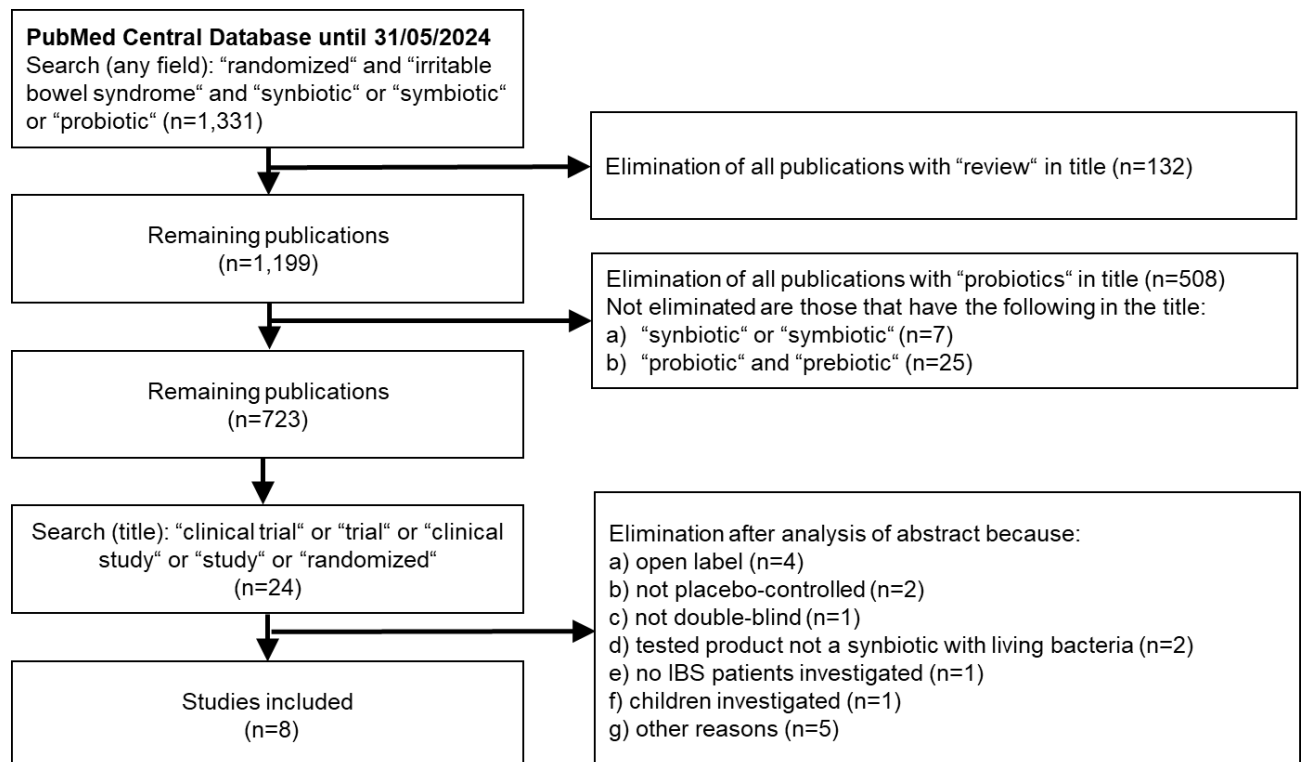

Supplement: Supplementary file 1 [file microorganisms-12-01493-s001.zip › microorganisms-3071319-supplementary.pdf]
